# Endometrial Cancer as a Familial Tumor: Pathology and Molecular Carcinogenesis (Review)

**DOI:** 10.2174/138920209787847069

**Published:** 2009-04

**Authors:** Kouji Banno, Megumi Yanokura, Yusuke Kobayashi, Makiko Kawaguchi, Hiroyuki Nomura, Akira Hirasawa, Nobuyuki Susumu, Daisuke Aoki

**Affiliations:** Department of Obstetrics and Gynecology, Keio University School of Medicine, Tokyo, Japan

**Keywords:** HNPCC, Endometrial cancer, DNA mismatch repair gene, *hMLH1*, *hMSH6.*

## Abstract

Some cases of endometrial cancer are associated with a familial tumor and are referred to as hereditary nonpolyposis colorectal cancer (HNPCC or Lynch syndrome). Such tumors are thought to be induced by germline mutation of the DNA mismatch repair (MMR) gene, but many aspects of the pathology of familial endometrial cancer are unclear and no effective screening method has been established. However, the pathology of endometrial cancer with familial tumor has been progressively clarified in recent studies. At present, about 0.5% of all cases of endometrial cancers meet the clinical diagnostic criteria for HNPCC. A recent analysis of the three MMR genes (*hMLH1, hMSH2 *and *hMSH6*) revealed germline mutations in 18 of 120 cases (15.0%) of endometrial cancer with familial accumulation of cancer or double cancer, with a frameshift mutation of the *hMSH6 *gene being the most common. Many cases with mutation did not meet the current clinical diagnostic criteria for HNPCC, indicating that familial endometrial cancer is often not diagnosed as HNPCC. The results suggest that the *hMSH6 *gene mutation may be important in carcinogenesis in endometrial cancer and germline mutations of the MMR gene may be more prevalent in cases associated with familial accumulation of cancer. An international large-scale muticenter study is required to obtain further information about the pathology of endometrial cancer as a familial tumor.

## INTRODUCTION

The incidence of endometrial cancer among malignant gynecological tumors has increased with lifestyle and environmental changes, and has reached almost half of all cases of uterine cancer in Western countries. The absolute number and prevalence of cases of endometrial cancer have increased worldwide and control of this cancer is an urgent task. However, many aspects of the mechanism of carcinogenesis and pattern of advancement are unclear. Environmental factors such as obesity and a high estrogen level are thought to play important carcinogenic roles, but a close association with hereditary disposition has also been suggested, since double cancer and an increased incidence of cancer in relatives are common in patients with endometrial cancer.

A high incidence of concomitant endometrial cancer has been shown in female patients with hereditary nonpolyposis colorectal cancer (HNPCC), a typical familial tumor that is inherited in an autosomal dominant manner, indicating the presence of HNPCC-associated familial endometrial cancer. HNPCC has a high risk of development of colorectal cancer, and accumulation of colorectal cancer in a family line was initially reported by Wartin *et al*. in 1913. Subsequent analysis of this family led Lynch to propose the disease concept of cancer family syndrome in 1971 [1, 2]. In 1990, the International Collaborative Group (ICG)-HNPCC established the following clinical diagnostic criteria for HNPCC, which are referred to as the classic Amsterdam Criteria: 1) HNPCC is diagnosed when 3 or more patients with histologically confirmed colorectal cancer are present in a family line and one is a first relative of the other two, 2) colorectal cancer develops over two generations, and 3) one case is diagnosed at younger than 50 years old [3].

## CLINICAL DIAGNOSTIC CRITERIA FOR HNPCC

Since the Amsterdam Criteria for HNPCC were proposed in 1991, several other diagnostic criteria, including the Japanese Criteria and Bethesda Criteria, have been published. The confusion caused by the different criteria was resolved by revision of the Amsterdam Criteria by the ICG-HNPCC in 1999, to give the new Amsterdam Criteria [4] (Table **1**). These criteria address endometrial cancer, small intestinal cancer, urethral cancer, and kidney cancer, in addition to the colorectal cancer included in the classic criteria. Cases not meeting the classic Amsterdam Criteria may meet the new Amsterdam Criteria, and this has resulted in an increased number of cases diagnosed as HNPCC. In addition, discovery of HNPCC has become possible through investigation of familial medical histories of endometrial cancer patients. The revision also recognized the importance of cooperation among gynecologists for identification of HNPCC. One concern with the new criteria is the omission of ovarian, breast and stomach cancer, which may also be associated with HNPCC.

## MECHANISM OF HNPCC CARCINOGENESIS AND ASSOCIATION WITH ENDOMETRIAL CANCER

**Fig. (1):**
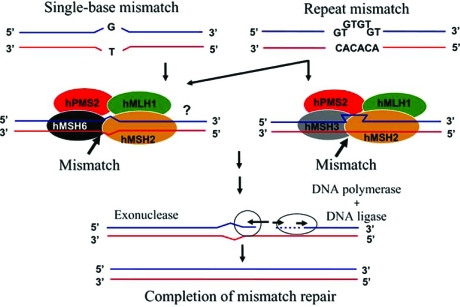
The DNA mismatch repair (MMR) mechanism in humans.

Six variants of the MMR gene, the causative gene in HNPCC, have been cloned: *hMSH2, hMLH1, hMSH3, hMSH6, hPMS1 *and* hPMS2*. An aberration in one of these genes prevents accurate repair of base mismatches produced during DNA replication, resulting in production of a DNA chain of altered length. This phenomenon is called microsatellite instability (MSI) and can lead to an increased frequency of errors in target genes involved in carcinogenesis, resulting in cancerization of the cell. Among the MMR genes, germline mutations of *hMLH1* on chromosome 3 and *hMSH2 *on chromosome 2 are thought to cause most cases of HNPCC, but it is unclear if these mutations are also causative for HNPCC-associated endometrial cancer. Mutation of *hMSH6* has also been proposed to be important for development of HNPCC-associated endometrial cancer, but the details are unclear (Fig. **1**) [5, 6].

The TGF-β type II receptor, which is involved in inhibition of cell proliferation, and BAX, which is associated with apoptosis induction, are candidate target genes in HNPCC [7, 8]. The *E2F* [9], *β-catenin* [10] and *TCF-4* [11] genes are also candidates, but the frequencies of aberration of these genes in endometrial cancer are low. Variation of the target gene involved in carcinogenesis in different organs has also been suggested. *PTEN* is also a commonly reported target gene in endometrial cancer [12], but its function remains unclear. Endometrial cancer develops through a mechanism similar to that of HNPCC, but may have unique biological characteristics. An analysis of endometrial cancer as a familial tumor has not been performed and many aspects of the cancer are unclear, including its genetic penetrance.

## CLINICAL CHARACTERISTICS OF HNPCC

**Fig. (2):**
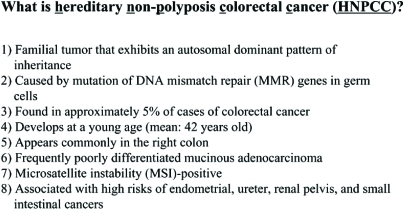
Clinical characteristics of HNPCC.

The frequency of HNPCC is unclear due to variation among reports, but HNPCC is thought to account for about 5% of all colorectal cancer cases [13-18] and to have the following clinicopathological characteristics: autosomal dominant inheritance; genetic penetrance of about 85% at 80 years of age; development at a young age; frequent development in the right colon; often poorly differentiated mucinous adenocarcinoma; diploid on cytometric analysis; marked intratumor infiltration of lymphocytes; microsatellite instability; high risk of development of endometrial cancer, urinary tract cancer, and small intestinal cancer; and a favorable prognosis [19]. The reason for the favorable prognosis despite many cases being poorly differentiated mucinous adenocarcinoma is unclear, but may be due to lymphocyte infiltration in the tumor and a low rate of lymph node metastasis. An association between aberration of MMR, the causative gene for HNPCC, and reduced sensitivity to anticancer drugs such as cisplatin has also been reported (Fig. **2**) [20].

## GYNECOLOGICAL CANCERS ASSOCIATED WITH HNPCC

The close association of endometrial cancer with HNPCC is apparent in the new Amsterdam Criteria. However, other gynecological cancers have also been reported in the first relatives of patients with HNPCC, with the incidence of endometrial cancer being highest at 9-19%, followed by stomach cancer and ovarian cancer at 6-14% and 5-7%, respectively [21]. An association of ovarian cancer with HNPCC has been suggested, but may not be as close as that of endometrial cancer, and a link between breast cancer and HNPCC has also attracted recent attention. Muir-Torre syndrome is an autosomal dominant hereditary disease of concomitant sebaceous gland tumors (adenoma, epithelioma and carcinoma) and malignant visceral tumors, and the incidences of colorectal and urogenital cancers are high among malignant visceral tumors, followed by breast cancer and malignant blood diseases. The lifetime incidence of breast cancer is about 12%. Mutation of the *hMSH2 *MMR gene has been found in 2 cases of Muir-Torre syndrome [22, 23] and this disease is assumed to have the pathology of hereditary breast cancer and to be a subtype of HNPCC.

## ENDOMETRIAL CANCER AND MICOSATELLITE INSTABILITY (MSI)

To investigate the status and clinicopathological characteristics of familial endometrial cancer, Banno *et al*. [24] surveyed the familial and medical histories of 385 patients who underwent treatment for endometrial cancer. MSI analysis was performed in 38 of these patients. The familial medical histories showed that 2 of the 385 cases met the new Amsterdam Criteria for HNPCC, giving a rate of HNPCC of about 0.5%. Investigation of familial accumulation of cancer in 890 relatives (439 men and 451 women) of the 38 endometrial cancer patients who underwent MSI analysis revealed high incidences of endometrial cancer, colorectal cancer and ovarian cancer, suggesting that a hereditary factor common to HNPCC is also involved in endometrial cancer. MSI analysis detected the presence of at least one of 5 microsatellite markers (D2S123, D3S1284, D5S404, D9S162, and hMSH2 intron 12) in 12 of the 38 cases (31.6%). This rate is very high compared to MSI in cancers of other organs, demonstrating that abnormal DNA mismatch repair plays an important role in endometrial cancer. The patients with MSI showed a tendency to have double cancer (such as ovarian cancer) compared with patients with microsatellite stability (MSS), although the difference was not significant (27% *vs*. 15%). Regarding prognosis, none of the MSI-positive cases were fatal (0/11, 0%), while 5 MSI-negative (MSS) cases were fatal (5/27, 19%). The difference was not significant, but this tendency is similar to that for HNPCC-associated colorectal cancer. The incidences of moderately differentiated adenocarcinoma G2 (36%) and poorly differentiated adenocarcinoma G3 (18%) tended to be higher in MSI-positive endometrial cancer, although the difference was not significant. These findings appear contradictory with the favorable prognosis, but interestingly they may reflect the biological characteristics of endometrial cancer induced by abnormal DNA mismatch repair [25].

## GERMLINE MUTATIONS IN ENDOMETRIAL CANCER PATIENTS WITH FAMILIAL ACCUMULATION OF CANCER

Hirai *et al*. [26] have recently performed an analysis of germline mutation of three MMR genes (*hMLH1, hMSH2 *and *hMSH6*) in 120 patients with endometrial cancer with strongly hereditary familial cancer accumulation or double cancer. Cases in which two or more first relatives had HNPCC-associated tumors (colorectal, endometrial, small intestinal, urethral, and renal pelvic cancers) and stomach, ovarian and breast cancers were designated as Group A, and cases with synchronous or metachronous double cancer of HNPCC-associated tumors (colorectal, endometrial, small intestinal, urethral and renal pelvic cancers) with stomach, ovarian or breast cancer were designated as Group B. Of the 120 cases, 57 were in Group A and 48 in Group B, with 15 in both groups. White blood cell-derived DNA was obtained from the patients and germline mutations were analyzed by direct sequencing of PCR products amplified using primers for all exons of *hMLH1, hMSH2 *and *hMSH6.* Germline mutation of the MMR genes was detected in 18 of the 120 cases (15.0%), including 9 of the 57 cases (15.8%) in Group A and 4 of the 48 cases (8.3%) in Group B. A high rate of germline mutation of MMR genes (5/15, 33.3%) was found in cases meeting the criteria for both groups. Mutations were detected in the *hMSH6 *gene in 9 cases (50%), the *hMLH1 *gene in 5, and the *hMSH2 *gene in 4. Mutations of the *hMSH6 *gene were concentrated in regions near exons 4-5 and were frameshifts in most cases. The mutations in 7 of the 18 cases had already been reported in the literature and registered in a mutation database, but those in the other 11 cases had not been reported previously [26] (Table **2**).

## SCREENING OF ENDOMETRIAL CANCER AS HNPCC

The clinical characteristics of HNPCC in colorectal cancer have gradually been clarified and HNPCC is thought to account for about 5% of all colorectal cancer cases. In contrast, there are only a few reports on endometrial cancer, despite its similar association with MMR gene aberration. An interview of families of 385 patients with endometrial cancer designed to investigate the familial medical histories of cancer revealed 2 patients who met the clinical diagnostic criteria for HNPCC [24]. This indicates that only 0.5% of all endometrial cancer cases are diagnosed as HNPCC, and this rate is markedly lower than that in colorectal cancer.

Microsatellite instability caused by MMR gene aberration is detectable by PCR using microsatellite markers. In screening for HNPCC, use of 5 microsatellite markers (BAT25, BAT26, D5S346, D2S123 and D17S250) is recommended. However, a germline mutation of the MMR gene was detected in only 1 of 12 patients with MSI-positive endometrial cancer (8.3%) and this mutation was a nonsense mutation at codon 100 of the *hMLH1 *gene. This mutation was already registered as a functional gene mutation in the Human Gene Mutation Database (HGMD), but this case did not meet the new Amsterdam Criteria for HNPCC [26]. This suggests that current clinical diagnostic criteria and MSI analysis have limited value in screening for HNPCC-associated endometrial cancer.

In Hirai *et al*. [26], germline mutation of the MMR gene was present in 15.8% of patients with endometrial cancer with familial accumulation of cancer, but not meeting the clinical diagnostic criteria for HNPCC. This suggests that MMR gene aberration should be kept in mind in management of patients with a tendency for familial cancer, regardless of the clinical assessment. In particular, familial development of breast, ovarian and stomach cancer, which are not recognized as HNPCC-associated cancers, should be investigated in interviews on familial medical histories. No germline mutation of the MMR gene was present in cases with double cancer alone without familial cancer accumulation, but all double cancers developed earlier than endometrial cancer, which is very interesting from the perspective of screening for endometrial cancer.

The *hMSH6* gene was the most commonly mutated MMR gene found by Hirai *et al*. [26], rather than *hMLH1 *or *hMSH2*, which are frequently mutated in colorectal cancer. This finding indicates the importance of *hMSH6* in development of endometrial cancer in HNPCC, and suggests an association between the MMR genotype and the cancer phenotype. Most interestingly, the mutations of *hMSH6 *were concentrated in exons 4-5 and were frameshifts. One of the *hMSH6 *mutations was a reported functional mutation, but the concentration in exons 4-5 differed from reports in Western patients. Thus, it is possible that this gene mutation is characteristic for HNPCC-associated endometrial cancer (Table **3**) [27, 28].

## FUTURE DIRECTIONS AND CONCLUSION

Many HNPCC-associated cases of endometrial cancer caused by MMR gene aberration do not meet the current clinical diagnostic criteria for HNPCC. Gene analysis focusing on the *hMSH6 *gene may allow efficient identification of familial endometrial cancer in cases of endometrial cancer with a tendency of familial accumulation of cancer. The findings that some cases of endometrial cancer are familial tumors and that an abnormal DNA mismatch repair gene is linked to development of the cancer are important for understanding the biological characteristics of endometrial cancer. Thus, it is important to identify and analyze patients with this type of endometrial cancer. However, cases of familial endometrial cancer that meet the current diagnostic criteria account for only 0.5% of all cases, and a close examination of family history is essential in screening for this cancer. An improved understanding of familial tumors among gynecologists and an international large-scale muticenter study are required for further progress, with establishment of a system of genetic tests and counseling for patients. There are currently many aspects of familial endometrial cancer that are unclear, but clarification of the pathology and development of a surveillance system and genetic tests are likely to produce new diagnostic and therapeutic methods for various types of endometrial cancer.

## Figures and Tables

**Table 1:** Clinical Diagnostic Criteria for HNPCC

**Amsterdam Minimum Criteria (1990)** At least 3 cases of colorectal cancer in relatives (verified pathologically) One is a first degree relative of the other two At least two successive generations should be affected One case of colorectal cancer diagnosed before the age of 50 years old FAP should be excluded
**Revised Amsterdam Criteria II (1998)** 1. At least 3 relatives with an HNPCC-associated cancer (cancer of the colorectum, endometrium, small bowel, ureter or renal pelvis) 2-5. As for the minimum criteria

**Table 2:** Eighteen Patients with Endometrial Cancer had MMR Gene Mutations in Germ Cells

No.	Age	Type	Grade	Stage	Group	Mutation Type	Report	Double Cancer
K007	42	EM/CL	G2	3c	AB	hMLH1 ex3 codon100 Nonsense	+	CC(42yo)
K009	42	EM	G1	1b	A	hMSH6 ex5 codon1085 Frameshift	+	−
K017	34	EM	G2	1b	A	hMSH2 ex10 codon554 Missense	−	−
K023	56	EM	G1	1a	A	hMSH6 ex4 codon795 Frameshift	−	−
K030	52	EM	G2	3a	A	hMSH6 ex4 codon546 Frameshift	−	−
K031	54	EM	G2	1a	A	hMSH2 ex11 codon582 Frameshift	−	−
T004	56	EM	G1	3a	A HNPCC	hMSH2 ex13 codon680 Nonsense	+	−
T011	65	AS	G1	3a	B	hMSH6 ex6 codon1163 Missense	−	BC(65yo)
T016	41	EM	G1	1b	AB HNPCC	hMSH2 ex5 codon288 Nonsense	+	CC(38,50yo)
J001	48	EM	G1	1b	AB HNPCC	hMLH1 ex16 codon618 Deletion	+	CC(44yo)
J015	53	EM	G1	1a	B	hMSH6 ex4 codon744 Frameshift	−	OvC(53yo)
J021	55	EM	G1	1b	A	hMSH6 ex4 codon482 Nonsense	−	−
G001	52	EM	G1	3c	AB HNPCC	hMLH1 ex13 Splicing	+	CC(48,50yo)
G030	57	EM	G2	1b	B	hMSH6 ex10 codon1355 Frameshift	−	BC(39yo)
G032	62	EM	G3	1a	A	hMSH6 ex4 codon487 Frameshift	−	−
G036	50	EM	G1	1a	A	hMSH6 ex4 codon742 Missense	−	−
F001	37	EM	G1	1a	B	hMLH1 ex12 codon384 Missense	−	CC(36yo)
F002	46	EM	G1	1b	AB	hMLH1 ex16 codon618 Deletion	+	CC(46yo)

CC: Colorectal Cancer, BC: Breast Cancer, OvC: Ovarian Cancer.

**Table 3:** MMR Gene Mutation in Germ Cells in Endometrial Cancer

Gene	Incidence of Germline Mutation in HNPCC	Incidence of Germline Mutation in HNPCC-Related Endometrial Cancer
*hMSH2*	+++	+
*hMSH3 *	−	?
*hMSH6 (GTBP)*	+	+++
*hMLH1*	+++	++
*hPMS1*	+	?
*hPMS2*	+	?

## References

[1] Lynch HT, Krush AJ (1971). The cancer family syndrome and cancer control. Surg. Gynecol. Obstet.

[2] Lynch HT, Lynch JF (2000). Hereditary nonpolyposis colorectal cancer. Semin. Surg. Oncol.

[3] Vasen HF, Mecklin JP, Khan PM, Lynch HT (1991). The International Collaborative Group on Hereditary Nonpolyposis Colorectal Cancer. Dis. Colon. Rectum.

[4] Vasen HF, Watson P, Mecklin JP, Lynch HT (1999). New clinical criteria for hereditary nonpolyposis colorectal cancer (HNPCC, Lynch syndrome) proposed by the International Colaboratory Group on HNPCC. Gastroenterology.

[5] Miyaki M, Konishi M, Tanaka K, Kikuchi-Yanoshita R, Muraoka M, Yasuno M, Igari T, Koike M, Chiba M, Mori T (1997). Germline mutation of MSH6 as the cause of hereditary nonpolyposis colorectal cancer. Nat Genet.

[6] Akiyama Y, Sato H, Yamada T, Nagasaki H, Tsuchiya A, Abe R, Yuasa Y (1997). Germline mutation of hMSH6/GTBP gene in an atypical hereditary nonpolyposis colorectal cancer kindred. Cancer Res.

[7] Markowitz S, Wang J, Myeroff L, Parsons R, Sun L, Lutterbaugh J, Fan RS, Zborowska E, Kinzler KW, Vogelstein B (1995). Inactivation of the Type II TGF-βreceptor in colon cancer cells with microsatellite instability. Science.

[8] Rompino N, Yamamoto H, Ionov Y, Li Y, Sawai H, Reed JC, Perucho M (1997). Somatic frameshift mutation in the BAX gene in colon cancers of the microsatellite mutator phenotype. Science.

[9] Ikeda M, Orimo H, Moriyama H, Nakajima E, Matsubara N, Mibu R, Tanaka N, Shimada T, Kimura A, Shimizu  K (1998). Close correlation between mutation of E2F and hMSH3 genes in colorectal cancers with microsatellite instability. Cancer Res.

[10] Miyaki M, Iijima T, Kimura J, Yasuno M, Mori T, Hayashi Y, Koike M, Shitara N, Iwama T, Kuroki T (1999). Frequent mutation of beta-catenin and APC gene in primary colorectal tumors from patients with hereditary nonpolyposis colorectal cancer. Cancer Res.

[11] Planck M, Wenngern E, Borg A, Olsson H, Nibert M (2000). Somatic frameshift alterations in mononucleotide repeat-coating genes in different tumor types from an HNPCC family with germline MSH2 mutation. Genes Chromosomes Cancer.

[12] Tashiro H, Blazes MS, Wu R, Cho KR, Bose S, Wang SI, Li J, Parsons R, Ellenson LH (1997). Mutation in PTEN are frequent in endometrial cancer but rare in other common gynecological malignancies. Cancer Res.

[13] Lynch HT, Smyrk TC, Watson P, Lanspa SJ, Lynch JF, Lynch PM, Cavalieri RJ, Boland CR (1993). Genetics, natural history, tumor spectrum, and pathology of hereditary nonpolyposis colorectal cancer. Gastroenterology.

[14] Moslein G, Tester DJ, Lindor NM, Honchel R, Cunningham JM, French AJ, Halling KC, Schwab M, Goretzki P, Thibodeau SN (1996). Microsatellite instability and mutation analysis of *hMSH2* and *hMLH1* in patients with sporadic, familial and hereditary colorectal cancer. Hum. Mol. Genet.

[15] Wu Y, Nyström-Lahti M, Osinga J, Looman MW, Peltomäki P, Aaltonen LA, de la Chapelle A, Hofstra RM, Buys CH (1997). *MSH2* and *MLH1* mutations in sporadic replication error-positive colorectal carcinoma as assessed by two-dimensional DNA electrophoresis. Genes Chromosomes Cancer.

[16] Herfarth K, Kodner IJ, Whelan AJ, Ivanovich JL, Bracamontes JR, Wells Sa Jr, Goodfellow PJ (1997). Mutations in *MLH1* are more frequent than in *MSH2* in sporadic colorectal cancers with microsatellite instability. Genes Chromosomes Cancer.

[17] Genuardi M, Anti M, Capozzi E, Leonardi F, Fornasarig M, Novella E, Bellacosa A, Valenti A, Gasbarrini GB, Roncucci L, Benatti P, Percesepe A, Ponz de Leòn M, Coco C, de Paoli A, Valentini M, Boiocchi M, Neri G, Viel A (1998). *MLH1* and *MSH2* constitutional mutations in colorectal cancer families not meeting the standard criteria for hereditary nonpolyposis colorectal cancer. Int. J. Cancer.

[18] Nomura S, Sugano K, Kashiwabara H, Taniguchi T, Fukayama N, Fujita S, Akasu T, Moriya Y, Ohhigashi S, Kakizoe T, Sekiya T (2000). Enhanced detection of deleterious and other germline mutations of *hMSH2* and *hMLH1* in Japanese hereditary nonpolyposis colorectal kindreds. Biochem. Biophys. Res. Commun.

[19] Horii A, Han HJ, Shimada M, Yanagisawa A, Kato Y, Ohta H, Yasui W, Tahara E, Nakamura Y (1994). Frequent replication errors at microsatellite loci in tumors of patients with multiple primary cancers. Cancer Res.

[20] Fink D, Nebel S, Aebi S, Zheng H, Cenni B, Nehmé A, Christen RD, Howell SB (1996). The role on DNA mismatch repair in platinum drug resistance. Cancer Res.

[21] Watoson P, Lynch HT (1994). The tumor spectrum in HNPCC. Anticancer Res.

[22] Kurse R, Rütten A, Lamberti C, Hosseiny-Malayeri HR, Wang Y, Ruelfs C, Jungck M, Mathiak M, Ruzicka T, Hartschuh W, Bisceglia M, Friedl W, Propping P (1998). Muir-Torre phenotype has a frequency of DNA mismatch-repair gene mutations similar to that in hereditary nonplyposis colorectal cancer families defined by the Amsterdam Criteria. Am. J. Hum. Genet.

[23] Bapat B, Xia L, Mandlensky L, Mitri A, Tonin P, Narod SA, Gallinger S (1996). The genetic basis of Muir-Torre syndrome includes hMLH1 locus. Am. J. Hum. Genet.

[24] Banno K, Susumu N, Hirao T, Yanokura M, Hirasawa A, Aoki D, Udagawa Y, Sugano K, Nozawa S (2004). Two Japanese kindreds occurring endometrial cancer meeting new clinical criteria for hereditary non-polyposis colorectal cancer (HNPCC), Amsterdam Criteria II. J. Obstet. Gynaecol. Res.

[25] Banno K, Susumu N, Yanokura M, Hirao T, Iwata T, Hirasawa A, Aoki D, Sugano K, Nozawa S (2004). Association of HNPCC and endometrial cancer. Int. J. Clin. Oncol.

[26] Hirai Y, Banno K, Suzuki M, Ichikawa Y, Udagawa Y, Sugano K, Miki Y (2008). Molecular epidemiological and mutation analysis of DNA mismatch repair (MMR) genes in endometrial cancer patients with HNPCC-associated familial predisposition to cancer. Cancer Sci.

[27] Berends MJ, Wu Y, Sijimons RH, Mensink RG, van der Sluis T, Hordijik-Hos JM, de Vries EG, Hollema H, Karrenbeld A, Buys CH, van der Zee AG, Hofstra RM, Kleibeuker JH (2002). Molecular and clinical characteristics of MSH6 variants: an analysis of 25 index carriers of germline variant. Am. J. Hum. Genet.

[28] Hampel H, Frankel W, Panescu J, Lockman J, Sotamaa K, Fix D, Comeras I, La Jeunesse J, Nakagawa H, Westman J.A, Prior T.W, Clendenning M, Penzone P, Lombardi J, Dunn P, Cohn DE, Copeland L, Eaton L, Fowler J, Lewandowski G, Vaccarello L, Bell J, Reid G, de la Chaelle A (2006). Screening for Lynch syndrome (hereditary nonpolyposis colorectal cancer) among endometrial cancer. Cancer Res.

